# Biological characterization of SN32976, a selective inhibitor of PI3K and mTOR with preferential activity to PI3Kα, in comparison to established pan PI3K inhibitors

**DOI:** 10.18632/oncotarget.17730

**Published:** 2017-05-09

**Authors:** Gordon W Rewcastle, Sharada Kolekar, Christina M Buchanan, Swarna A Gamage, Anna C Giddens, Kit Y Tsang, Jackie D Kendall, Ripudaman Singh, Woo-Jeong Lee, Greg C Smith, Weiping Han, David J Matthews, William A Denny, Peter R Shepherd, Stephen M.F Jamieson

**Affiliations:** ^1^ Auckland Cancer Society Research Centre, School of Medical and Health Sciences, The University of Auckland, Auckland, New Zealand; ^2^ Maurice Wilkins Centre for Molecular Biodiscovery, The University of Auckland, Auckland, New Zealand; ^3^ Department of Molecular Medicine and Pathology, School of Medical and Health Sciences, The University of Auckland, Auckland, New Zealand; ^4^ Department of Pharmacology, School of Medical Sciences, The University of New South Wales, Sydney, Australia; ^5^ Laboratory of Metabolic Medicine, Singapore Bioimaging Consortium, Agency for Science, Technology and Research (A*STAR), Singapore, Singapore; ^6^ PharmIntuition, San Francisco, CA, USA; ^7^ Department of Pharmacology and Clinical Pharmacology, School of Medical and Health Sciences, The University of Auckland, Auckland, New Zealand

**Keywords:** SN32976, phosphatidylinositol 3-kinase, pan PI3K inhibitor, PI3Kα-preferential, kinase selectivity

## Abstract

Multiple therapeutic agents have been developed to target the phosphatidylinositol 3-kinase (PI3K) signaling pathway, which is frequently dysregulated in cancer promoting tumor growth and survival. These include pan PI3K inhibitors, which target class Ia PI3K isoforms and have largely shown limited single agent activity with narrow therapeutic windows in clinical trials. Here, we characterize SN32976, a novel pan PI3K inhibitor, for its biochemical potency against PI3K isoforms and mTOR, kinase selectivity, cellular activity, pharmacokinetics, pharmacodynamics and antitumor efficacy relative to five clinically-evaluated pan PI3K inhibitors: buparlisib, dactolisib, pictilisib, omipalisib and ZSTK474. SN32976 potently inhibited PI3K isoforms and mTOR, displaying preferential activity for PI3Kα and sparing of PI3Kδ relative to the other inhibitors, while showing less off-target activity than the clinical inhibitors in a panel of 442 kinases. The major metabolites of SN32976 were also potent PI3K inhibitors with similar selectivity for PI3Kα as the parent compound. SN32976 compared favorably with the clinically-evaluated PI3K inhibitors in cellular assays, inhibiting pAKT expression and cell proliferation at nM concentrations, and *in* animal models, inducing a greater extent and duration of pAKT inhibition in tumors than pictilisib, dactolisib and omipalisib at similarly tolerated dose levels and inhibiting tumor growth to a greater extent than dactolisib and ZSTK474 and with similar efficacy to pictilisib and omipalisib. These results suggest that SN32976 is a promising clinical candidate for cancer therapy with enhanced kinase selectivity and preferential inhibition of PI3Kα compared to first generation pan PI3K inhibitors, while retaining comparable anticancer activity.

## INTRODUCTION

The phosphatidylinositol 3-kinase (PI3K) signaling pathway regulates a number of crucial cellular processes, including growth, survival, angiogenesis, migration and metabolism [1, 2, 3, 4]. PI3K signaling is frequently dysregulated in cancer resulting in persistent pathway activation [5, 6]. Activating mutations in the *PIK3CA* oncogene that encodes the p110α catalytic subunit of class Ia PI3K are common in a range of cancers, particularly breast, uterine, cervical and colorectal cancers, while mutations in other class I PI3K genes (*PIK3CB*, *PIK3CD*, *PIK3CG*) are less common [7, 8]. Genetic or epigenetic inactivation of the negative regulator of PI3K activity, the tumor suppressor *PTEN*, is found in a number of cancers [9]. Several other genes downstream of PI3K are also frequently mutated, such as *AKT* genes, *MTOR*, *TSC1* and *TSC2* [7]. In addition to activating mutations, amplification of genes encoding PI3K/AKT enzymes or receptor tyrosine kinases (e.g. EGFR, HER2, KIT, PDGFRα, MET) can also promote PI3K signaling [6].

Since the PI3K pathway is so frequently dysregulated in many cancer types, a large number of therapeutic agents have been developed to target PI3K enzymes. These include drugs that target multiple class Ia PI3K isoforms and mTOR as well as compounds that selectively target individual class I PI3K isoforms [10]. One of these, idelalisib, an inhibitor of PI3Kδ has been approved for use in chronic lymphocytic leukemia, relapsed follicular B-cell non-Hodgkin's lymphoma and relapsed small lymphocytic lymphoma. Selective inhibitors of PI3Kα, such as alpelisib and serabelisib, are in phase II and III trials having shown promise in combination with standard therapies in ER-positive breast cancer [11] and head and neck squamous cell carcinoma [12], while GSK2636771, a PI3Kβ inhibitor, is in phase II trials after showing activity in PTEN deficient tumors [13] and IPI-549, a PI3Kγ inhibitor, is in phase I trials on account of its ability to prevent immune suppression in solid tumors [14].

Currently, the most advanced pan PI3K inhibitor in clinical development is buparlisib. This drug is being tested in a large number of clinical trials (88 trials registered with the NIH; 37 ongoing), both as a single agent and part of combination therapy, on the basis of promising preclinical activity [15]. Despite showing limited single agent activity [16], improved patient outcomes have been observed to date in hormone receptor-positive breast cancer [17, 18] and head and neck squamous cell carcinoma [19] in combination with other therapies, but treatment has been associated with serious adverse effects, including a rise in liver enzymes and severe anxiety and depression [18]. Several other pan PI3K and PI3K/mTOR inhibitors have entered clinical development including pictilisib, copanlisib, ZSTK474, omipalisib and dactolisib, but many of these have now been discontinued due to toxicity and limited efficacy. There is therefore a clear need for inhibitors of PI3K with improved therapeutic windows [20, 21].

**Figure 1:**
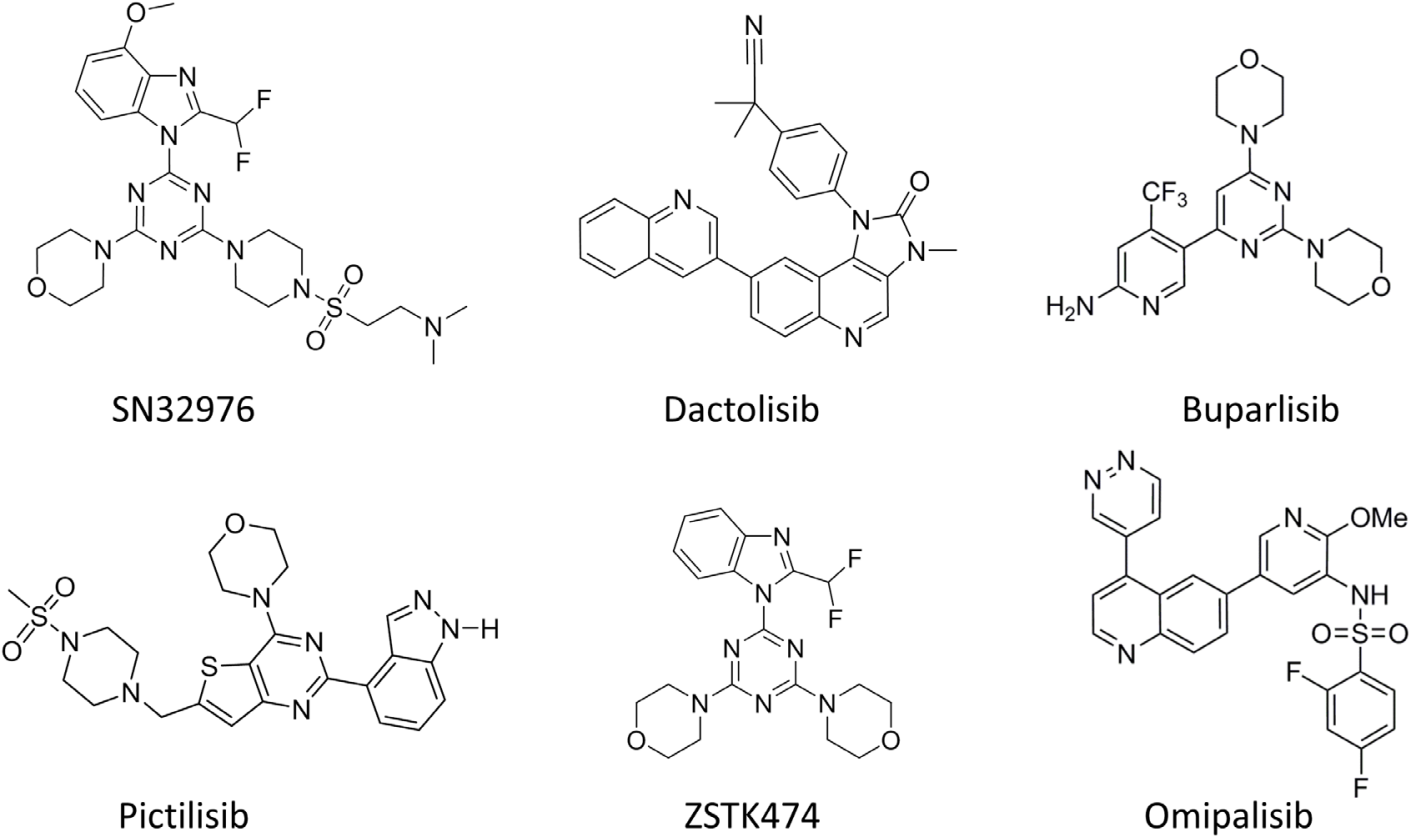
Chemical structures of SN32976, dactolisib, buparlisib, pictilisib, ZSTK474 and omipalisib

Here, we report on a novel PI3Kα-preferential pan PI3K inhibitor SN32976, providing a biological characterization of its preclinical activity. Moreover, we compare its preclinical activity to that of five other pan PI3K inhibitors that have progressed into clinical trial: buparlisib (BKM120), dactolisib (BEZ235), pictilisib (GDC0941), omipalisib (GSK2126458) and ZSTK474 (Figure 1).

## RESULTS

### PI3K/mTOR biochemical activity and kinase selectivity

SN32976, 2-((4-(4-(2-(difluoromethyl)-4-methoxy-1*H*-benzo[*d*]imidazol-1-yl)-6-morpholino-1,3,5-triazin-2-yl)piperazin-1-yl)sulfonyl)-*N, N*-dimethylethan-1-amine, was developed, based on an earlier scaffold [22], to be a potent and selective inhibitor of PI3K enzymes with favorable pharmacological properties. The design and synthesis of SN32976 will be reported elsewhere (manuscript in preparation). The biochemical potency of SN32976 against the major class I PI3K isoforms and mTOR was determined against purified recombinant protein and compared to five PI3K/mTOR inhibitors that have been clinically evaluated: ZSTK474, dactolisib, pictilisib, buparlisib and omipalisib (Table 1). SN32976 is a potent inhibitor of class I PI3K enzymes and mTOR with similar activity to PI3Kα, β, γ and mTOR to that of ZSTK474, dactolisib, pictilisib and buparlisib, but less potency than omipalisib. Of particular interest, however, was the relative sparing of SN32976 to PI3Kδ and selectivity for PI3Kα. SN32976 had an IC_50_ of 15.1 ± 4.3 nM against PI3Kα and showed 7.3-fold, 8.9-fold, 13-fold and 30-fold selectivity against PI3Kγ, PI3Kδ, mTOR and PI3Kβ, respectively. None of the other pan PI3K inhibitors showed more than 2-fold selectivity for PI3Kα over all other PI3K isoforms and mTOR.

**Table 1:** Biochemical IC_50_ values for the PI3K inhibitors against class I PI3K enzymes and mTOR

IC_50_ (nM)	PI3Kα^a^	PI3Kβ^a^	PI3Kγ^a^	PI3Kδ^a^	mTOR^b^
SN32976	15.1 ± 4.3	461 ± 195	110 ± 41	134 ± 20	194 ± 55
Pictilisib	39.3 ± 2.8	217 ± 38	182 ± 40	26.0 ± 3.4	298 ± 85
ZSTK474	13.1 ± 1.1	50.6 ± 9.5	82.7 ± 27.2	16.5 ± 3.2	102 ± 19
Buparlisib	162 ± 32	607 ± 19	1042 ± 75	582 ± 9	293 ± 161
Dactolisib	15.8 ± 5.8	313 ± 107	87.5 ± 2.5	26.0 ± 7.2	2.8 ± 0.3
Omipalisib	0.61 ± 0.22	1.8 ± 0.5	1.2 ± 0.2	0.55 ± 0.22	2.6 ± 0.4

^a^ IC_50_ values (mean ± SEM, n≥2) determined by HTRF assay.

^b^ IC_50_ values (mean ± SEM, n=2) determined by Invitrogen.

The six PI3K/mTOR inhibitors were screened for selectivity against a panel of 442 kinases (Supplementary Table 1). At 1 μM, SN32976 and buparlisib showed high selectivity for the class I PI3K enzymes, mTOR and mutant forms of PIK3CA with no other kinases showing >80% inhibition (Supplementary Figure 1). Pictilisib was similarly selective for PI3K and mTOR at 1 μM, with significant off-target inhibitory activity only against the pseudokinase JH2 domain of JAK1 (89%). The other compounds were less selective at 1 μM: ZSTK474 had high binding affinity for the class II PI3K enzymes PIK3C2B and PIK3C2G as well as the G2019S mutant of LRRK2 (99.7%), dactolisib showed >80% inhibition to CLK1, CLK4, ERBB3, FLT3 (D835Y mutant), the catalytic JH1 domains of JAK1 and JAK2, MAP4K2, PIK3C2B and PIK3C2G, and omipalisib was non-selective against PIK3C2B, PIK3C2G and PIK4CB. Due to their enhanced selectivity at 1 μM, SN32976, pictilisib and buparlisib were also tested at 10 μM. Again, SN32976 showed a high level of selectivity, with off-target activity only against PIK3C2B and PIK3C2G (Supplementary Figure 2). Buparlisib also remained selective at 10 μM, but bound to CLK1, CLK2 and CLK4 with >80% affinity, while pictilisib had >80% off-target binding at 10 μM to 34 kinases other than the class I PI3K enzymes, mTOR and mutant PIK3CA.

### *In vitro* inhibition of phosphorylated AKT expression and cell proliferation

**Figure 2:**
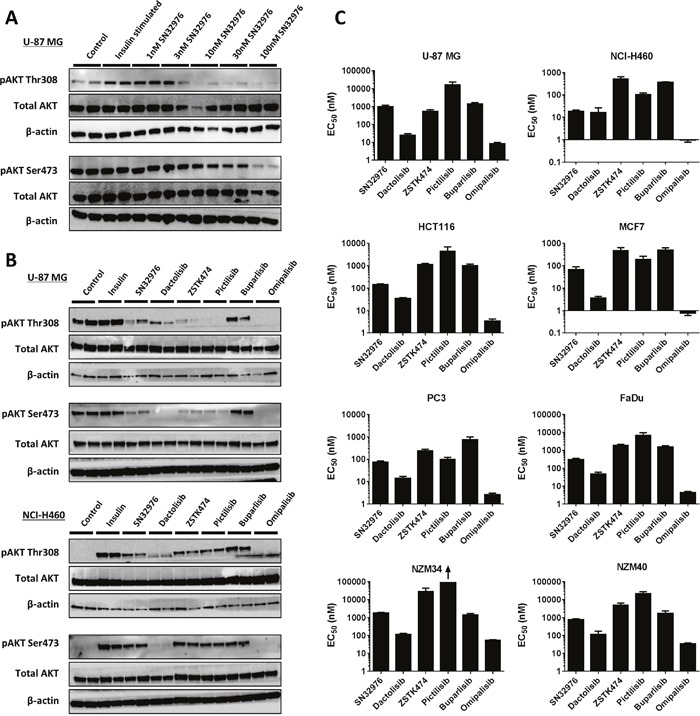
SN32976 inhibits pAKT expression and cell proliferation similarly to other PI3K inhibitors **(A)** pAKT and total AKT expression in U-87 MG cells after treatment with multiple concentrations of SN32976 for 1 h and **(B)** in U-87 MG and NCI-H460 cells after treatment with different PI3K inhibitors at 100 nM for 15 min. Cells were serum starved overnight prior to treatment and stimulated with 500 nM insulin for 5 min. **(C)** EC_50_ values for PI3K inhibitors at preventing cell proliferation in 8 different cell lines with dysregulated PI3K signaling. Bars represent the mean and standard error of n=3-6 separate determinations. The arrow indicates that the EC_50_ was above the highest drug concentration tested. The bars above the blots in **(A)** and **(B)** indicate that duplicate cultures were tested with each compound.

To determine if SN32976 could prevent PI3K signaling in cells, phosphorylated AKT (pAKT) expression was investigated as a biomarker of PI3K activity in U-87 MG (PTEN null) and NCI-H460 (*PIK3CA* E545K mutant) cells. SN32976 inhibited both Thr308 and Ser473 pAKT expression in U-87 MG cells at concentrations as low as 10 nM (Figure 2A). The effect of SN32976 on pAKT expression in U-87 MG and NCI-H460 cells was greater than the inhibition of pAKT induced by buparlisib, particularly in U-87 MG cells, and comparable to the inhibitory activity of ZSTK474 and pictilisib (Figure 2B). Dactolisib, as a result of its greater potency against mTOR, was more active against Ser473 pAKT, while omipalisib induced greater inhibition of both phosphorylation sites of AKT, due to its greater potency against the PI3K and mTOR enzymes.

Since PI3K/mTOR signaling is involved in the regulation of cell proliferation, we next investigated whether SN32976 could inhibit cell proliferation across a panel of cell lines with dysregulated PI3K signaling. The cell lines were PTEN null (U-87 MG, PC3, NZM34), H1047R *PIK3CA* mutant (HCT116, NZM40), E545K *PIK3CA* mutant (NCI-H460, MCF7) and *PIK3CA* amplified (FaDu). SN32976 potently inhibited cell proliferation in all cell lines, with EC_50_ values ranging from 18.5 ± 4.7 nM in NCI-H460 cells to 1787 ± 318 nM in NZM34 cells (Figure 2C). The EC_50_ values compared favorably with those of the other pan PI3K inhibitors, with equal or lower EC_50_ values observed with SN32976 compared to ZSTK474, pictilisib and buparlisib across the cell line panel. However, the greatest potencies at inhibiting cell proliferation were observed with dactolisib and omipalisib, most likely related to their enhanced mTOR inhibition. Overall the pattern of cell proliferation inhibition for the 6 inhibitors was highly consistent across the 8 cell lines.

### ADME and plasma pharmacokinetics of SN32976

We evaluated the ADME properties of SN32976 to ensure it was a suitable candidate for *in vivo* testing. SN32976 was highly soluble at low pH, was moderately bound to plasma proteins and had reasonable stability in liver microsomes (Table 2). It did not inhibit any of the major CYP isoforms at 20 μM, caused minimal induction of CYP1A2 and CYP3A4, was non-mutagenic and only inhibited hERG at high concentrations.

**Table 2:** ADME properties of SN32976

Properties	Value (mean ± SD)
Solubility: pH 2.0/7.4 (μM)	>100,000/11.2 ± 0.3
Plasma protein binding: human/mouse/rat/dog (% bound)	96.5 ± 0.4/90.5 ± 0.6/90.0 ± 0.7/89.1 ± 2.7
Liver microsomes: human/mouse/rat/dog (% remaining at 30 min)	32.6 ± 0.2/27.1 ± 2.0/38.1 ± 0.3/47.5 ± 1.1
CYP inhibition: 1A2/2C9/2C19/2D6/3A4 IC_50_ (μM)	>25/20.0 ± 5.5/>25/>25/>25
CYP induction at 10 μM: 1A2/2B6/3A4	1.27 ± 0.01/0.31 ± 0.01/1.39 ± 0.21
hERG IC_50_ vs I_Kr_ current (μM)	≥30
Ames: Salmonella (TA97a, TA98, TA100, TA1535), E. Coli (WP2 uvrA pKM101)	Non-mutagenic

**Figure 3:**
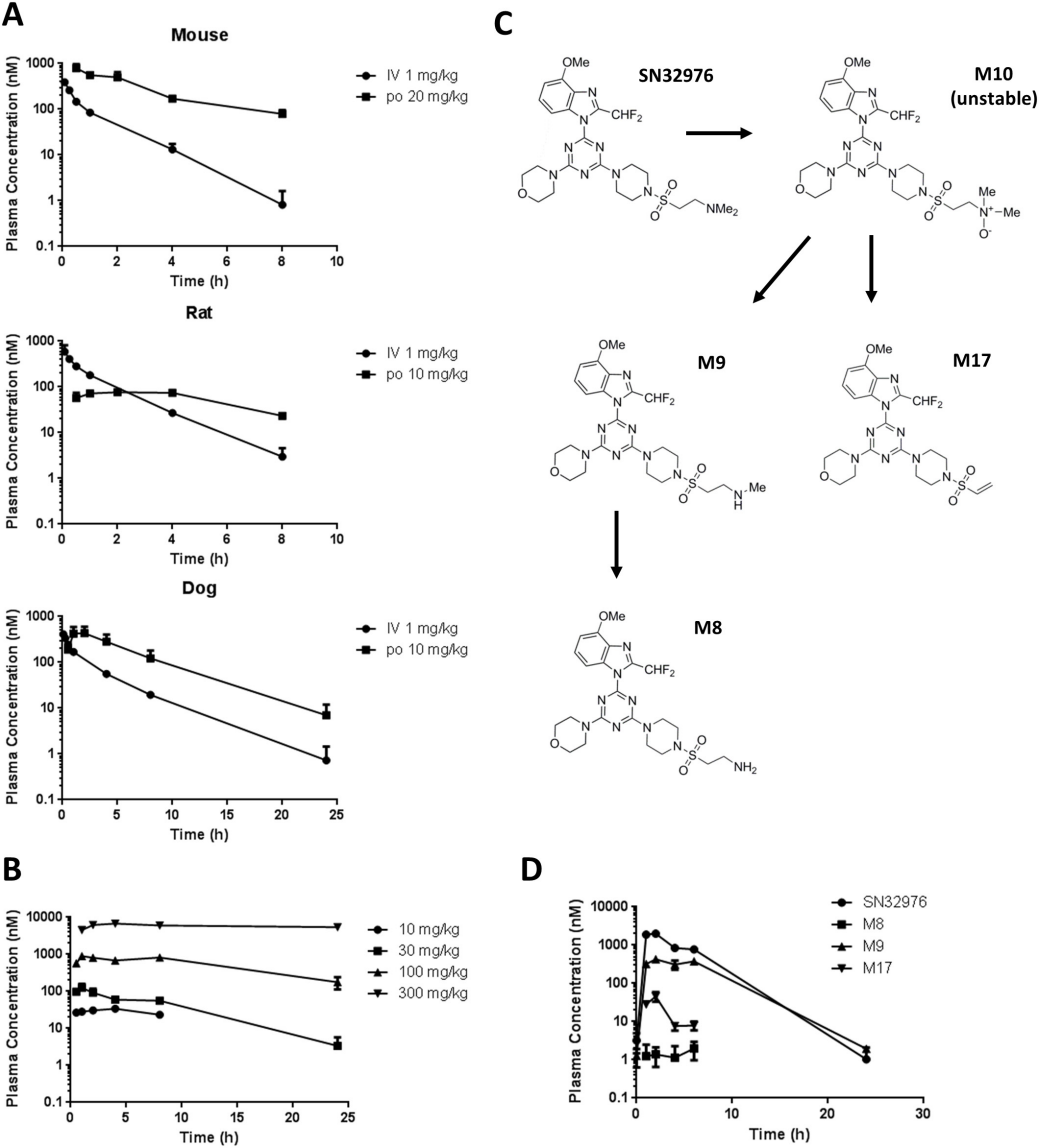
Plasma pharmacokinetics of SN32976 and its major metabolites **(A)** Plasma pharmacokinetics of SN32976 after IV and oral (po) dosing in mice, rats and dogs. **(B)** Plasma pharmacokinetics of multiple dose levels of oral SN32976 in rats. **(C)** Purported metabolism scheme of SN32976 to generate the major metabolites in liver microsomes. **(D)** Plasma pharmacokinetics of SN32976 and stable major metabolites in mice treated with 30 mg/kg SN32976 by oral gavage. Symbols and error bars represent the mean and standard error from 3 separate animals.

The plasma pharmacokinetics of SN32976 were determined in mice, rats and dogs to identify if suitable concentrations of parent drug could be achieved in plasma *in vivo*. Following oral doses of 10 or 20 mg/kg, sufficient area under the curve (AUC) values were obtained (2504 nM.h and 3224 nM.h) to give suitable oral bioavailability (33.4% and 35.9%) values in mice and dogs, respectively, to support the use of oral dosing in subsequent *in vivo* studies (Figure 3A) (Supplementary Table 2). Low AUC (579 nM.h) and bioavailability (8.1%) values were obtained after 10 mg/kg oral dosing in rats; however, increased doses revealed non-linear pharmacokinetics in rats after oral dosing, such that high AUC values and much longer half-lives were observed (Figure 3B) (Supplementary Table 3).

### Identification of active metabolites of SN32976

Incubation of SN32976 in mouse, rat, dog, monkey and human liver microsomes revealed the presence of 17 metabolites of SN32976 with m/z of between 530-615 (Supplementary Figure 3). The majority of these metabolites were found in either trace (<1% relative peak areas in the total ion chromatograph) or minor (1-10% relative peak areas) quantities in liver microsomes. However, 4 metabolites were found to be major (>10% relative peak areas) in at least one species. The putative structures were elucidated for the metabolites based on their molecular weights to provide a proposed biotransformation scheme for SN32976 (Figure 3C). SN32976 is thought to first undergo N-oxidation to give the unstable metabolite M10. This is then further degraded by deamination and dehydration to give M17 or by demethylation to give M9. M9 can be further N-demethylated to give M8. M9 and M17 were found to be major metabolites in liver microsomes of all species, while M8 was major in monkey microsomes, minor in human, trace in dog and not present in rat or mouse microsomes. M10 was major in mouse and rat, minor in monkey and dog and trace in human microsomes.

To determine if the stable major metabolites of SN32976 are active against PI3K enzymes, M8, M9 and M17 were synthesized and tested for biochemical potency against PI3Kα, β, γ, δ and mTOR (Table 3). All 3 metabolites were active inhibitors of PI3K enzymes and mTOR, displaying greater potency against PI3Kα than SN32976 (PI3Kα IC_50_ = 5.8 ± 0.4, 8.2 ± 1.8 and 6.2 ± 0.5, for M8, M9 and M17, respectively). The metabolites retained moderate PI3Kα selectivity with M8, M9 and M17 showing 6.5-fold, 5.6-fold and 4.2-fold selectivity, respectively, for PI3Kα over other PI3K isoforms and mTOR. In addition, M17 lacked activity against PI3Kβ.

**Table 3:** Class I PI3K and mTOR IC_50_ values for SN32976 and its major metabolites

IC_50_ (nM)	PI3Kα^a^	PI3Kβ^a^	PI3Kγ^a^	PI3Kδ^a^	mTOR^b^
SN32976	15.1 ± 4.3	461 ± 195	110 ± 41	134 ± 20	194 ± 55
M8	5.8 ± 0.4	108 ± 27	68.0 ± 18.6	37.6 ± 13.5	156 ± 44
M9	8.2 ± 1.8	61.5 ± 21.2	90.3 ± 17.1	45.8 ± 17.7	244 ± 56
M17	6.2 ± 0.5	>1000	26.3 ± 2.6	55.0 ± 24.1	74.9 ± 25.1

^a^ IC_50_ values (mean ± SEM, n≥3) determined by HTRF assay.

^b^ IC_50_ values (mean ± SEM, n=2) determined by Invitrogen.

Since the major metabolites of SN32976 can also inhibit PI3K enzymes and mTOR, we evaluated the plasma concentrations of the metabolites and the parent compound after SN32976 dosing in mice to determine their contribution to the pharmacokinetics of SN32976 (Figure 3D). Overall the combined AUC of SN32976 and the 3 stable major metabolites was 19942 nM.h, of which the parent compound contributed 71.8%, M8 0.13%, M9 27.1% and M17 1.0%.

### Pharmacokinetic-pharmacodynamic relationship of SN32976

**Figure 4:**
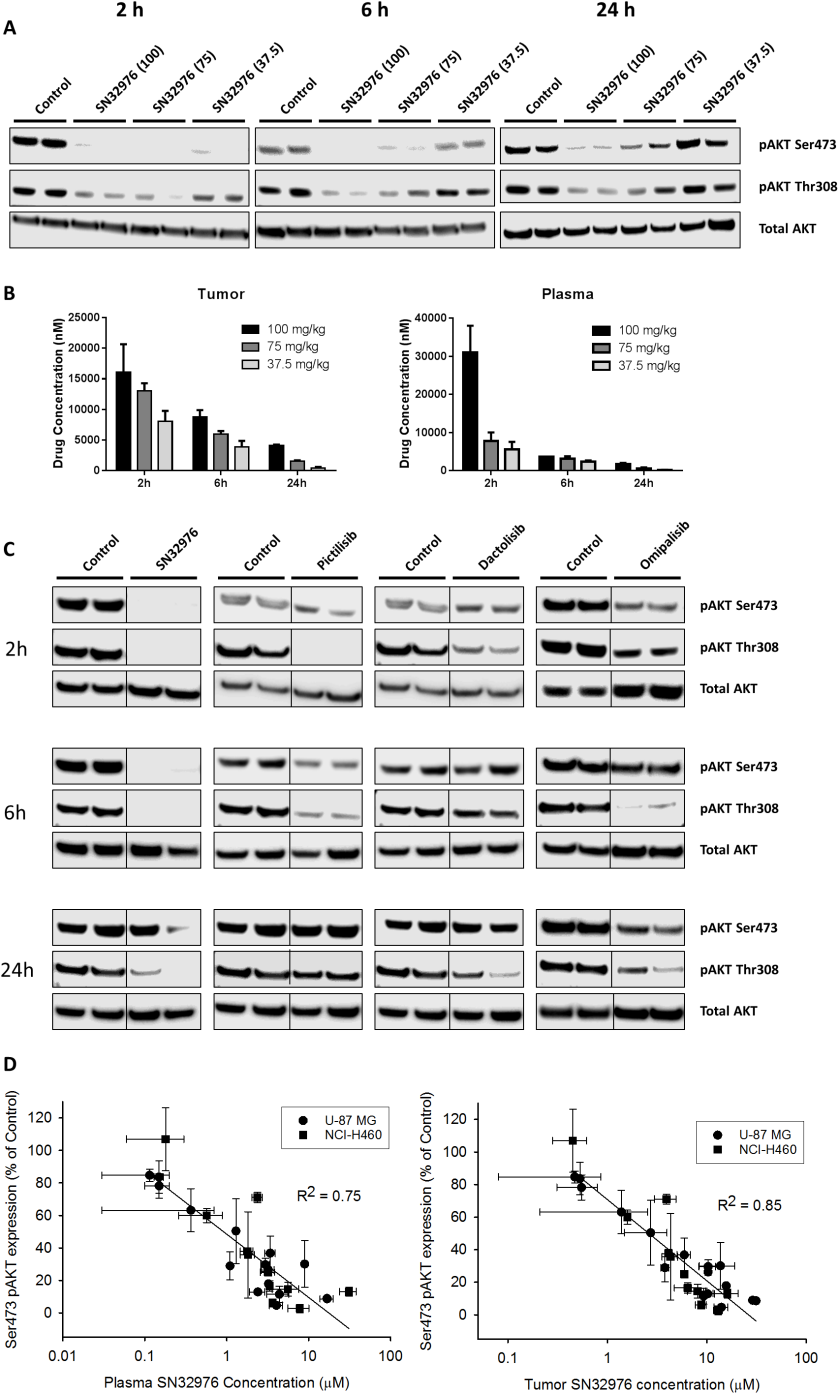
Pharmacokinetic-pharmacodynamic relationship for SN32976 **(A)** pAKT and total AKT expression in NCI-H460 tumors after single dose treatment in mice with multiple dose levels of SN32976. Mice were culled and tumors collected at the indicated timepoints after dosing. **(B)** Tumor and plasma concentrations of SN32976 in tumor and blood samples collected from mice bearing NCI-H460 tumors at the indicated timepoints after a single dose of 37.5, 75 or 100 mg/kg SN32976. **(C)** pAKT and total AKT expression in U-87 MG tumors 2, 6 and 24 h after single dose treatment in mice with 100 mg/kg SN32976, 100 mg/kg pictilisib, 25 mg/kg dactolisib or 1 mg/kg omipalisib. **(D)** Correlation between Ser473 pAKT expression in U-87 MG and NCI-H460 tumors and plasma or tumor SN32976 concentrations in the same mice at 2, 6 and 24 h after treatment at multiple dose levels of SN32976. Bars or symbols represent the mean and standard error from 2 separate animals. The bars above the blots in **(A)** and **(C)** indicate that duplicate cultures were tested with each compound or at each dose level.

The pharmacokinetic-pharmacodynamic (PK/PD) relationship of SN32976 was investigated to determine if the achievable plasma and tumor concentrations could result in pAKT inhibition *in vivo*. NIH-III mice with *PIK3CA*-mutant NCI-H460 tumors were treated with a single dose of SN32976 and plasma and tumor were collected at multiple timepoints after dosing for pharmacokinetic analysis and evaluation of pAKT expression. SN32976 at well tolerated doses substantially inhibited the expression of pAKT (both Ser473 and Thr308) for 24 h at 100 and 75 mg/kg and for approx. 6 h at 37.5 mg/kg (Figure 4A). Parent drug concentrations in the plasma and tumor were dose-dependent and were still present 24 h after dosing (Figure 4B). Similarly, in mice with PTEN-null U-87 MG tumors, multiple dose levels of SN32976 inhibited pAKT expression for up to 24 h after dosing, with 100 mg/kg inducing greater knockdown of pAKT than pictilisib, dactolisib or omipalisib at their maximum tolerated dose levels (Figure 4C and Supplementary Figure 4). The SN32976 data from the NCI-H460 and U-87 MG tumors were pooled to determine if there was a robust relationship between plasma or tumor SN32976 concentrations and pAKT expression. PK/PD analysis revealed that pAKT inhibition had a linear-log correlation with SN32976, with stronger correlations observed for Ser473 pAKT (plasma: R^2^=0.75, P<0.0001, Pearson Correlation; tumor: R^2^ = 0.85, P<0.0001) (Figure 4D) than for Thr308 pAKT (plasma: R^2^=0.43, P<0.001; tumor: R^2^ = 0.57, P<0.0001) (Supplementary Figure 5), and for tumor SN32976 concentrations over plasma drug concentrations. Overall, greater inhibition of pAKT was observed with increased plasma and tumor concentrations of SN32976, such that plasma concentrations in excess of approx. 1 μM and tumor concentrations in excess of approx. 2 μM were required to achieve 50% inhibition of ser473 and Thr308 pAKT expression *in vivo*.

### Effects of SN32976 on glucose metabolism

**Figure 5:**
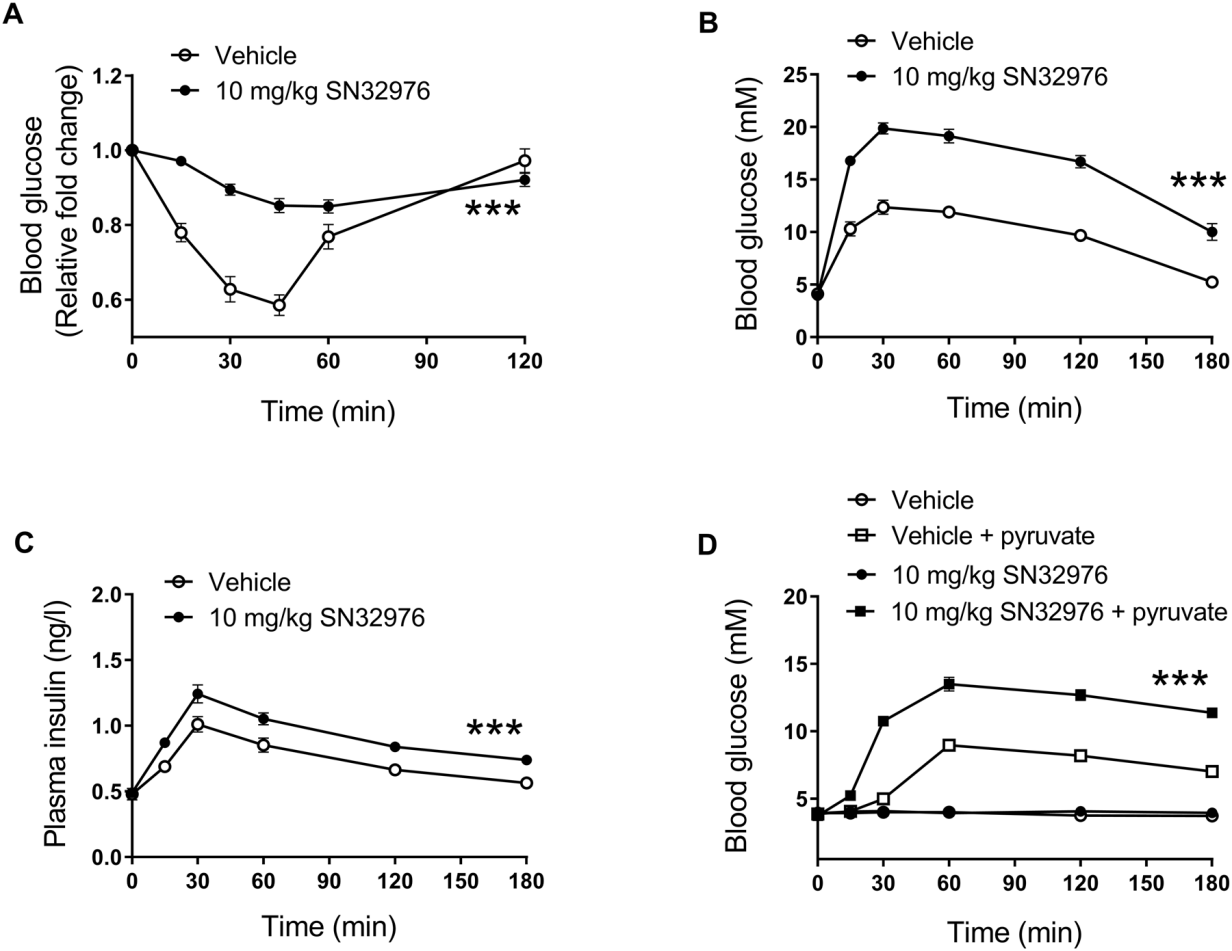
SN32976 alters glucose metabolism *in vivo* Male CD1 mice were pretreated for 1 h with 10 mg/kg SN32976 or drug vehicle by ip injection. **(A)** Blood glucose levels at multiple timepoints after animals were injected with 0.75 units/kg insulin in an insulin tolerance test. **(B)** Blood glucose and **(C)** insulin levels at multiple timepoints after animals were injected with 2 g/kg glucose in glucose tolerance tests. **(D)** Blood glucose levels at multiple timepoints after animals were injected with 2 g/kg pyruvate in a pyruvate tolerance test. Symbols represent the mean and standard error of ≥6 animals. Statistical significance was determined by repeated measures 2-way ANOVA (***, *P*<0.001 compared with controls).

Insulin's effects on glucose metabolism require PI3Kα activity and so impairments in glucose metabolism are a known on-target effect for drugs that target PI3Kα [3]. In line with this, pretreatment with 10 mg/kg SN32976 for 1 h significantly impacted the ability of mice to tolerate exogenous 0.75 U/kg insulin (Figure 5A) or 2 g/kg glucose (Figure 5B), with insulin levels also significantly increased following glucose administration (Figure 5C). Furthermore, SN32976 increased the hepatic production of glucose from 2 g/kg pyruvate (Figure 5D). These results provide further pharmacodynamic evidence for *in vivo* on-target efficacy for SN32976.

### Antitumor efficacy of SN32976 relative to other PI3K inhibitors

**Figure 6:**
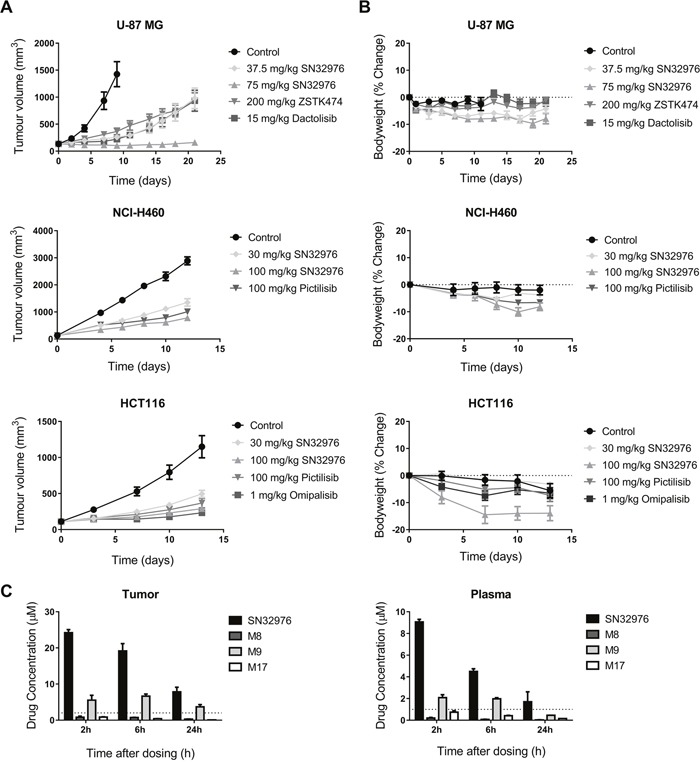
SN32976 prevents tumor growth at tolerated dose levels **(A)** Tumor volume in U-87 MG, NCI-H460 and HCT116 tumor xenograft models after daily treatment with SN32976, ZSTK474, dactolisib, pictilisib or omipalisib. Symbols represent the mean and standard error for 7-10 mice. **(B)** Bodyweight change in animals following daily treatment with SN32976, ZSTK474, dactolisib, pictilisib or omipalisib. **(C)** Tumor and plasma concentrations of SN32976 and its major metabolites in animals with U-87 MG tumors (n=2-3). Dotted lines represent the concentrations of SN32976 required to achieve approx. 50% inhibition of pAKT.

To evaluate the *in vivo* antitumor efficacy of SN32976, immunodeficient mice were inoculated with U-87 MG (PTEN-null), NCI-H460 and HCT116 (both *PIK3CA*-mutant) cells and treated daily with SN32976 and ZSTK474, dactolisib, pictilisib or omipalisib once tumors had established. SN32976, at doses up to 100 mg/kg, inhibited tumor growth to a similar or enhanced extent compared to the other PI3K inhibitors in the three tumor models (Figure 6A). SN32976 was well tolerated at the doses tested, although bodyweight loss did exceed 10% at 100 mg/kg in mice with HCT116 tumors (Figure 6B). Tumor and plasma SN32976 parent and major metabolite concentrations were determined in mice with U-87 MG tumors after multiple doses of 100 mg/kg SN32976. Tumor and plasma concentrations of SN32976 were maintained above the concentrations required to achieve 50% inhibition of pAKT for 24 h after dosing (Figure 6C). The relative levels of major metabolites in the plasma (AUC = 2.0%, 33.6% and 8.8% of parent for M8, M9 and M17, respectively) and tumor (AUC = 3.7%, 32.9% and 2.4% of parent for M8, M9 and M17, respectively) were largely consistent with our earlier determination in plasma after SN32976 dosing at 30 mg/kg; however, M8 was found to preferentially distribute to tumor, where it was detected at higher levels than M17.

## DISCUSSION

Signaling through the PI3K/mTOR pathway is frequently upregulated in cancer, making this pathway a common therapeutic target for anticancer agents. A large number of agents have been developed that target individual or all class I PI3K enzymes and/or mTOR, with several of these progressing into clinical development [10]. Here, we describe the biological characterization of the highly selective and PI3Kα-preferential pan PI3K/mTOR inhibitor SN32976 and compare its activity directly to a number of pan PI3K inhibitors that have entered clinical development, including the Novartis compound buparlisib that has been registered in 88 clinical trials to date. SN32976 was developed as a solubilized analogue of earlier PI3K inhibitors [22] through the addition of a (2-dimethylaminoethyl)sulfonamide group. SN32976 compared favorably to the clinically evaluated pan PI3K inhibitors in terms of its PI3Kα-selectivity, kinase selectivity, pharmacokinetic-pharmacodynamic properties and antitumor efficacy, suggesting that it has potential as a clinical candidate.

SN32976 inhibits all class I PI3K enzymes and the closely related kinase mTOR, but shows greatest activity against the PI3Kα isoform. We determined the biochemical activity of SN32976 and five clinically evaluated PI3K inhibitors under the same assay conditions to allow direct comparisons of the data. The IC_50_ values we generated were similar to those previously reported for these inhibitors [23, 24, 25, 26, 27]. SN32976 had similar potency against PI3K isoforms and mTOR to the clinical PI3K inhibitors, with the exception of omipalisib, which was more potent against all enzymes and dactolisib, which showed enhanced activity against mTOR. However, unlike the other inhibitors, which showed similar potency against PI3Kα and PI3Kδ or mTOR, SN32976 showed overall selectivity towards PI3Kα (at least 7-fold over all other PI3K isoforms and mTOR). Sparing of PI3Kδ by SN32976 may increase its therapeutic window by preventing toxicities associated with PI3Kδ inhibition such as inhibition of regulatory T-cells [28], which is thought to be responsible for the severe hepatotoxicity in patients treated with the PI3Kδ inhibitor idelalisib [29]. Despite showing selectivity for PI3Kα, SN32976 is still active against other PI3K isoforms and mTOR and is less selective than PI3Kα-selective inhibitors, such as alpelisib and serabelisib, which have >50-fold selectivity for PI3Kα over other class I PI3K isoforms and mTOR [30, 31]. Therefore, we consider SN32976 as a PI3Kα preferential pan PI3K inhibitor rather than a PI3Kα-selective inhibitor, but acknowledge that the degree of selectivity observed for PI3Kα may change with different assay conditions (i.e. with variations in concentrations of ATP or PI3K enzymes).

In addition to its preferential activity against PI3Kα, SN32976 was highly selective for class I PI3K enzymes, mTOR and mutant forms of PIK3CA in a panel of 442 kinases, while the other inhibitors, especially dactolisib at 1 μM and pictilisib at 10 μM, all showed off-target binding to other kinases. This off-target activity as well as on-target activity against PI3Kδ has likely contributed to the narrow therapeutic windows that have resulted in a lack of efficacy in clinical trials for these agents [20, 32, 33, 21] leading to the discontinuation of dactolisib and pictilisib and providing evidence that newer more selective inhibitors of PI3K are required to improve tolerability and increase efficacy [20, 21]. All of the inhibitors, except buparlisib, were active against the class II PI3K enzymes PIK3C2B and PIK3C2G (10 μM only for SN32976 and pictilisib), which despite a limited understanding can regulate cellular processes, such as invasion and migration and their inhibition may contribute to the anticancer activity of these agents, rather than just off-target toxicity [34, 35]. It is notable that the pan PI3K inhibitor that has shown the greatest clinical activity, buparlisib [17, 18, 19], was the only agent other than SN32976 to show kinase selectivity and greatest potency against PI3Kα, yet it was >10-fold less potent against PI3Kα than SN32976 and showed only 1.8, 3.6 and 3.7-fold selectivity over mTOR, PI3Kδ and PI3Kβ, respectively.

Importantly, despite its reduced activity against PI3Kδ and PI3Kγ relative to PI3Kα, SN32976 compared favorably with the pan PI3K inhibitors in cellular assays and animal models. In a range of cell lines, SN32976 inhibited pAKT expression and cell proliferation at nM concentrations with similar or better activity to pictilisib, buparlisib and ZSTK474, but less potency than omipalisib and dactolisib, presumably due to their enhanced mTOR inhibition. As observed in U-87 MG cells compared to NCI-H460 cells, the knockdown in pAKT expression did not always translate to inhibition of cell proliferation, reflecting the different timescales of these assays (15 min vs 120 h incubation). SN32976 also impaired glucose metabolism, indicating its ability to target PI3Kα *in vivo*, with effects consistent with those previously observed with dactolisib and ZSTK474 [3]. Furthermore, the extent and duration of pAKT inhibition in tumors was greater for SN32976 than for pictilisib, dactolisib and omipalisib at similarly tolerated dose levels, while SN32976 inhibited tumor growth to a greater extent than dactolisib and ZSTK474 and with similar efficacy to pictilisib and omipalisib. This data is consistent with the literature reports for the clinical PI3K inhibitors [36, 25, 27, 15, 37, 38], and shows that SN32976 has at least comparable preclinical activity with these inhibitors in *PIK3CA*-mutant and PTEN-null cells and tumors, suggesting that the reduced potency of SN32976 against PI3Kβ, δ and γ does not appear to influence its preclinical anticancer activity. In comparison, PI3Kα-selective inhibitors typically require *PIK3CA* mutations or combination therapies to be highly active [39, 40], suggesting that maintaining some, but not necessarily high, activity against PI3Kβ, δ, γ and/or mTOR is essential to retain anticancer activity in the absence of *PIK3CA* mutations. Therefore, by preferentially targeting PI3Kα, but still inhibiting other PI3K isoforms and mTOR, SN32976 may combine the improved tolerance of PI3Kα-selective inhibitors with the enhanced anticancer efficacy of pan PI3K inhibitors.

The 3 most prevalent stable metabolites identified in liver microsomes and mouse plasma were all active inhibitors of PI3K, with slightly greater potency against PI3Kα and similar PI3Kα-selectivity to the parent compound. These metabolites were most likely produced through demethylation or deamination of the dimethylamino substituent group of SN32976 that was added to enhance solubility rather than potency or selectivity, so it is not surprising that the metabolites retained PI3Kα activity and selectivity. Of these metabolites, the N-mono-methylated metabolite M9 was detected at levels of 33-38% of the parent compound in plasma and U-87 MG tumors and therefore likely contributed to the anticancer efficacy of SN32976 *in vivo*. The M8 and M9 metabolites also appeared to be more stable than the parent in U-87 MG tumors, suggesting that they may help prolong the antitumor activity of SN32976. That the metabolites retain similar PI3Kα-selectivity to the parent ensures that off-target effects are limited and that dosing with SN32976 has a sufficient therapeutic window to delay tumor growth at well tolerated dose levels.

In general, therapeutic targeting of PI3K signaling has focused on agents that target individual or all isoforms, sacrificing either single agent efficacy or tolerability in the process. Novel approaches have seen the use of isoform-sparing PI3K inhibitors, such as taselisib, which targets PI3Kα, δ, γ but spares PI3Kβ [41] and is in phase III trials for metastatic breast cancer, having shown antitumor activity in earlier trials [42, 43]. In SN32976, we believe we have identified a second generation pan PI3K inhibitor with greater kinase selectivity than the first generation inhibitors as well as enhanced PI3Kα-selectivity relative to the other class I PI3K and mTOR isoforms. We expect that SN32976 will have reduced off-target effects due to its clean kinase profile as well as reduced on-target toxicity, due to its PI3Kα-selectivity. However, by maintaining some activity against PI3Kβ, δ, γ and mTOR, SN32976 is highly effective at preventing tumor growth in preclinical tumor models. We believe this data supports the progression of SN32976 into clinical development.

## MATERIALS AND METHODS

### Compounds

SN32976 and metabolites [44, 45], dactolisib [46, 47], pictilisib [24], ZSTK474 [22, 38], buparlisib [23] and omipalisib [25] were synthesized at the Auckland Cancer Society Research Centre using methods previously described. For *in vivo* studies, pictilisib was synthesized as the bismethanesulfonate salt [48] and SN32976 as both hydrochloride and methanesulfonate salts [45].

### *In vitro* kinase assays

The biochemical activity of the drug compounds against PI3Kα, PI3Kβ, PI3Kγ, and PI3Kδ was determined using the PI3K (human) HTRF assay (Millipore) as described previously [40]. Each PI3K enzyme was titrated and used at a concentration between its EC_65_ and EC_80_. ATP was added at its approximate Km value of 10 μM. mTOR activity was determined through the SelectScreen kinase profiling service (Invitrogen) using the Z’LYTE kinase assay. Compound selectivity against related protein or lipid kinases was determined using the scanMAXX KINOMEscan (Ambit Biosciences) of 442 kinases.

### Cell culture

Cell lines were obtained from ATCC, except PC3 (supplied by Dr Ronnie Cohen) and NZM34 and NZM40 (generated from New Zealand melanoma patients) [49]. The cell lines were maintained in α-modified minimal essential medium (ThermoFisher Scientific) supplemented with 5% fetal calf serum (FCS; Moregate Biotech) (HCT-116, NCI-H460, FaDu), 5% FCS with 1% penicillin-streptomycin (ThermoFisher Scientific) and ITS (5 μg/ml insulin, transferrin and 5 ng/ml sodium selenite; Roche) (NZM34 and NZM40), 5% FCS with 1% penicillin-streptomycin, ITS and 5 ng/ml epidermal growth factor (Sigma-Aldrich) (MCF-7), 10% FCS (U-87 MG) or 10% FCS and 1% penicillin-streptomycin-glutamine (PC-3). Cell lines were authenticated by short tandem repeat profiling of extracted DNA at DNA Diagnostics Ltd and were kept in culture for no longer than 6 months. NZM34 and NZM40 were cultured under 5% O_2_.

### Cell proliferation and western blotting

Cells were seeded into 96-well plates at 400 – 5000 cells/well and left to settle for 2 h at 37 °C with 5% CO_2_ (and 5% O_2_ for NZM34 and NZM40). The plates were incubated with compounds at a range of concentrations in 0.2% or less DMSO for 5 days before fixing in 10% trichloroacetic acid and staining with 0.4% sulforhodamine B as described previously [47]. U-87 MG and NCI-H460 cells were seeded into 12-well plates at approx. 500,000 cells/well and were left to settle for 24 h at 37 °C with 5% CO_2_. Cells were serum starved overnight then treated with drug compound for 15 min – 1 h, prior to stimulation with 500 nM insulin for 5 min. Cell lysates were prepared and blotted for pAKT (Ser473, #9271, 1:1000; Thr308, #9275, 1:1000; Cell Signaling Technology), total AKT (#9272, 1:1000, Cell Signaling Technology) and β-actin (A1978, 1:2000, Sigma-Aldrich) as described previously [47]. Quantitation of pAKT and total AKT staining was carried out by densitometry analysis using ImageJ 1.40g (National Institutes of Health, USA).

### ADME/toxicity studies

Solubility, plasma protein binding, microsomal stability (Seventh Wave), cytochrome P450 induction/inhibition (Cyprotex), hERG testing (IPS Therapeutique) and Ames testing (WIL Research) of SN32976 were carried out by contract research organizations. Brief descriptions of each assay are provided below, with detailed protocols available from the individual companies. Solubility of SN32976 was determined by adding compound to 100 mM potassium phosphate buffer (pH 7.4) with 1 mM MgCl_2_ adjusted to pH 7.4 or 2.0 until no further compound dissolved. Plasma protein binding was determined by adding 1 μM SN32976 to 10% human, dog, mouse or rat plasma separated from PBS (pH 7.4) by an equilibrium dialysis membrane and incubating for 16-24 h at 37°C at 500 rpm. Microsomal stability was determined in human, mouse, rat and dog liver microsomes (0.5 – 1 mg/ml; ThermoFisher) incubated with SN32976 at 1 μM in the presence of 2 mM NADPH for up to 60 min at 37°C. All solubility, plasma protein binding and liver microsomes samples were quantitatively analyzed by LC-MS/MS. Cytochrome P450 inhibition of CYP1A2, CYP2C9, CYP2C19, CYP2D6 and CYP3A4 was evaluated in human liver microsomes incubated with SN32976 and quantitated by LC-MS/MS or ethoxyresorufin fluorescence (CYP1A2). Cytochrome P450 induction was evaluated in cultured hepatocytes incubated with multiple concentrations of SN32976 for 72 h prior to qRT-PCR determination of mRNA expression of CYP1A2, CYP2B6 and CYP3A4. The effect of SN32976 on the rapidly activating delayed-rectifier potassium selective current (IKr) was evaluated in HEK 293 cells transfected with the hERG gene by patch-clamp assay. For Ames testing, SN32976 was evaluated for frameshift and base-pair substitution mutagenicity at concentrations up to 250 μg/well in four *Salmonella* tester strains (TA97a, TA98, TA100 and TA1535) and one *Escherichia Coli* strain (WP2 *uvrA* pKM101) in the presence and absence of rat liver S9 fraction.

### Animals and dosing

Male CD1 mice (6-8 weeks old; Charles River), male Sprague-Dawley rats (7-9 weeks old; Charles River) and male Beagle dogs (8-24 months old; Marshall Farms) were used for plasma pharmacokinetic studies. Tumor pharmacokinetic, pharmacodynamic and antitumor efficacy studies were carried out in 6-8 week old female balb/c nude (Beijing HFK Bioscience) or female balb/c Rag1^−/−^ (Vernon Jansen Unit, the University of Auckland) mice inoculated with 5×10^6^ U-87 MG, NCI-H460 or HCT116 cells in PBS. Male CD1 mice (6-8 weeks old) were used for glucose metabolism studies. All animal experimentation followed approved protocols from relevant animal ethics committees with animals having ad libitum access to food and water. Animals were treated with the hydrochloride or mesylate salts of SN32976 (at the free base equivalent) formulated in 5% dextrose in water. For the rat dose proportionality study, SN32976 was formulated in Hot Rod Chemistry formulation #6 (HRC #6; Pharmatek). ZSTK474 was administered in 2% carboxymethylcellulose (Sigma-Aldrich) with 1% Tween-80. Dactolisib, pictilisib and omipalisib were formulated in HRC #6 for pharmacodynamic studies and in 50% PEG-400 (Sigma-Aldrich) in 20% 2-hydroxypropyl-β-cyclodextrin (Sigma-Aldrich), 5% dextrose in water and 20% 2-hydroxypropyl-β-cyclodextrin, respectively for antitumor efficacy studies. Compounds were administered as a single IV or oral dose for pharmacokinetic and pharmacodynamic analysis or by oral gavage at qdx14 or qdx21 dosing schedules for evaluation of antitumor efficacy. SN32976 was administered by IP injection for glucose metabolism tests. All animals were monitored daily for signs of toxicity or weight loss after dosing and were culled if moderate signs of toxicity developed or if body weight loss exceeded 20% of pre-treatment weight. For tumor studies, animals were randomly stratified into treatment groups and dosing was initiated once tumors reached approx. 250 mm^3^ for pharmacodynamic analysis or 150 mm^3^ for antitumor efficacy. Tumor volumes were calculated from electronic caliper measurements of tumor diameter using the formula (L x w^2^) x π/6 (where L is the longest tumor diameter and w is the perpendicular diameter).

### *In vivo* pharmacology

Blood and tumor samples were collected at multiple time points after dosing (n=2-3/time point). Blood was collected into lithium-heparin or K_2_-EDTA tubes (Becton-Dickinson) and processed to plasma by centrifugation at 6000 rpm for 5 min. Methanol was added to plasma for protein extraction. Tumor samples were snap-frozen in liquid nitrogen, pulverized using a BioPulverizer (BioSpec Products), and added to methanol. Quantitative analysis together with an internal standard was performed by LC-MS/MS either on a Sciex API 4000 triple quadrupole LC-MS/MS (Applied Biosystems) with Prominence HPLC (Shimadzu) or Agilent 6460 LC-MS/MS using multiple reaction monitoring and electrospray ionization. Plasma and tumor drug concentrations were quantified against a calibration curve of standard concentrations in the relevant matrix ranging from 2 – 10000 nM and compared to quality control standards at 3 different concentrations. Pharmacokinetic parameters were calculated using Phoenix WinNonlin v6.2 (Pharsight Corp.) software. Tumor tissue lysates were prepared by adding lysis buffer to pulverized tumor tissue and centrifuging at 13000 rpm for 10 min at 4°C. Protein concentration was determined, and lysates blotted for pAKT, total AKT and β-actin as described previously [47]. Pharmacokinetic-pharmacodynamic correlations were determined by Pearson product moment correlation analysis in SigmaPlot 13.0 (Systat Software).

### Metabolite identification

Identification of SN32976 metabolites was determined in liver microsomes isolated from pooled human donors, male CD1 mice, male Sprague-Dawley rats, male Beagle dogs and male Cynomolgus monkeys (1 mg/ml; ThermoFisher). Liver microsomes were incubated with 10 μM SN32976 in the presence of 2 mM NADPH for 60 min at 37°C. The reaction was quenched by mixing samples with acetonitrile and internal standard. The samples were analyzed on a Sciex 4000 QTRAP LC-MS/MS linked to a SPD-10AV UV/Vis detector (Shimadzu) operating at dual wavelengths of 220 nm and 254 nm. Observation of parent compound and metabolites was achieved using electrospray ionization while performing a mass scan from 300-750 m/z. For observed peaks, the m/z was determined and product ion scans were carried out.

### Glucose metabolism analysis

Insulin tolerance tests, glucose tolerance tests and pyruvate tolerance tests were carried out in male CD-1 mice treated with SN32976, as described previously [3]. Statistical significance of differences between means was determined by repeated measures 2-way ANOVA using Prism 7.00 (GraphPad Software Inc.).

## SUPPLEMENTARY MATERIALS FIGURES AND TABLES





## Abbreviations

ATP: adenosine triphosphate
AUC: area under the curve
CYP: cytochrome P450
DMSO: dimethyl sulfoxide
EDTA: ethylenediaminetetraacetic acid
FCS: fetal calf serum
HTRF: homogeneous time-resolved fluorescence
IP: intraperitoneal
ITS: insulin, transferrin and selenium
IV: intravenous
LC-MS/MS: liquid chromatography with tandem mass spectroscopy
mTOR: mammalian target of rapamycin
m/z: mass to charge ratio
NADPH: nicotinamide adenine dinucleotide phosphate hydrogen
PBS: phosphate buffered saline
PI3K: phosphatidylinositol 3-kinase
PK/PD: pharmacokinetic pharmacodynamic relationship
po: oral
PTEN: phosphatase and tensin homolog
qRT-PCR: quantitative reverse transcription polymerase chain reaction
