# Supplementary material for: Biological characterization of SN32976, a selective inhibitor of PI3K and mTOR with preferential activity to PI3Kα, in comparison to established pan PI3K inhibitors
Source: Oncotarget. 2017 May 9;8(29):47725–40. doi: 10.18632/oncotarget.17730 (PMC5564600; doi:10.18632/oncotarget.17730)
Supplement: Supplementary file 1 [file oncotarget-08-47725-s001.pdf]

Biological characterization of SN32976, a selective inhibitor of PI3K and mTOR with preferential activity to PI3Kα, in comparison to established pan PI3K inhibitors

SUPPLEMENTARY TABLES AND FIGURES

Supplementary Table 1: Kinase selectivity data for the PI3K inhibitors

See Supplementary File 1

Supplementary Table 2: Pharmacokinetic parameters of SN32976

Compound[a]		IV					po				
		Dose (mg/kg)	Vd (L/kg)	Cl L/h/kg)	AUC (nM.h)	$T_{1/2}$ (h)	Dose (mg/kg)	C_{max} (nM)	AUC (nM.h)	$T_{1/2}$ (h)	F (%)
Mouse	SN32976.Cl	1	6.76	4.58	375	1.02	20	789	2504	2.37	33.4
Rat	SN32976.Cl	1	4.10	2.40	717	1.19	10	77.0	579	3.28	8.1
Dog	SN32976.MS	1	8.67	1.85	927	3.24	10	430	3224	3.79	35.9

[a] SN32976 was administered either as a hydrochloride salt (Cl) or a mesylate salt (MS).

Supplementary Table 3: Dose proportionality of SN32976 in rats

Dose[a]	C_{max} (nM)	AUC (nM.h)	$T_{1/2}$ (h)	T_{max} (h)	Dose proportionality	
					Actual[b]	Expected[c]
10 mg/kg	34.1	417	2.27	4	1	1
30 mg/kg	125	1067	4.62	1	2.6	3
100 mg/kg	893	16385	9.77	1	39.3	10
300 mg/kg	6808	138514	-	4	332.2	30

[a] SN32976 was administered as the mesylate salt by oral gavage.
[b] AUC relative to the AUC at 10 mg/kg.
[c] If AUC increased linearly with dose.

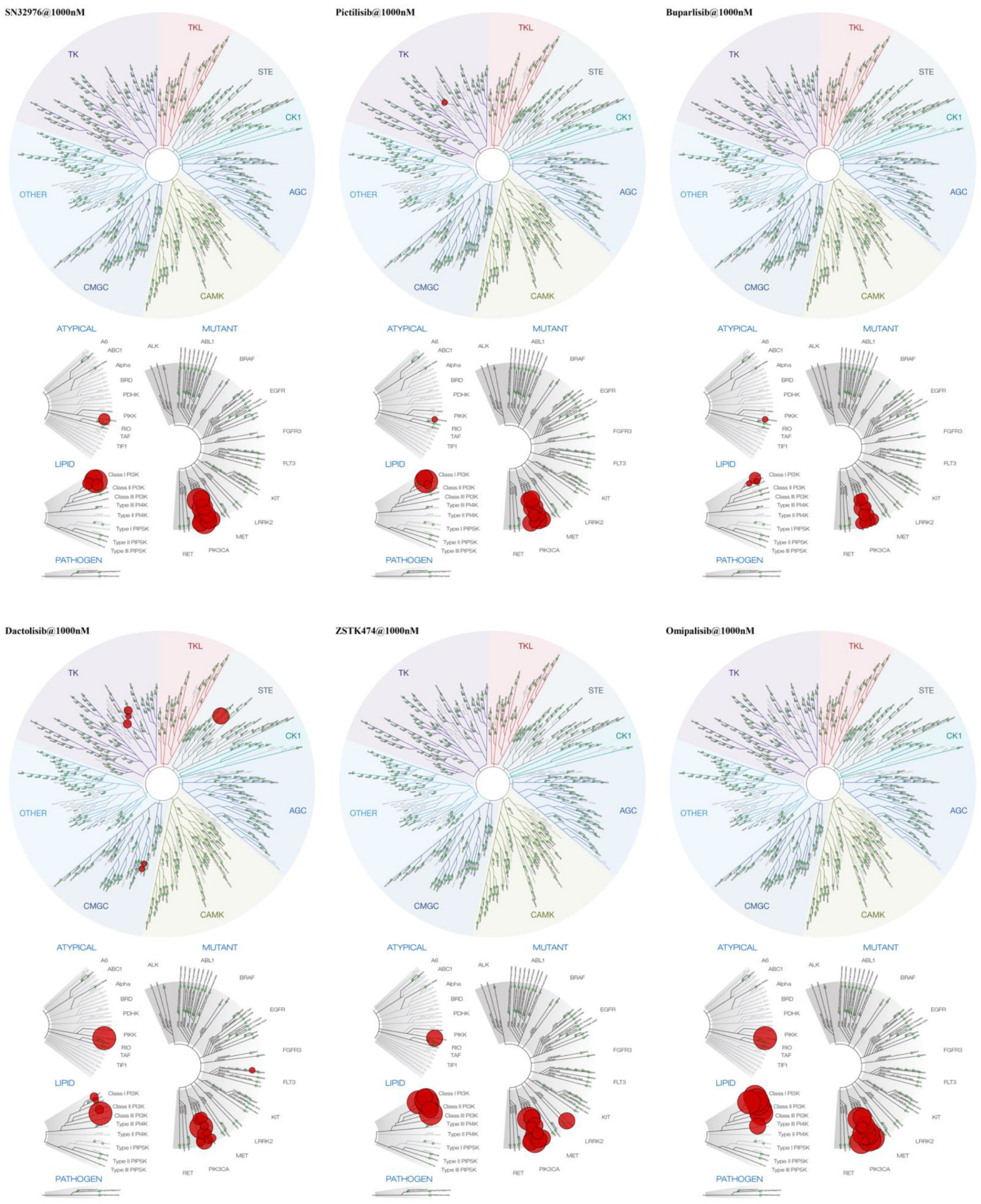

Supplementary Figure 1: Kinase dendrogram plots to visualize kinase interactions in the presence of 1 µM concentrations of each PI3K inhibitor. Kinases that bind at >80% are marked with red circles, with larger circles indicating higher-affinity binding. Images generated using TREE*spot*™ Software Tool and reprinted with permission from KINOME*scan*®, a division of DiscoveRx Corporation, ©DISCOVERX CORPORATION 2010.

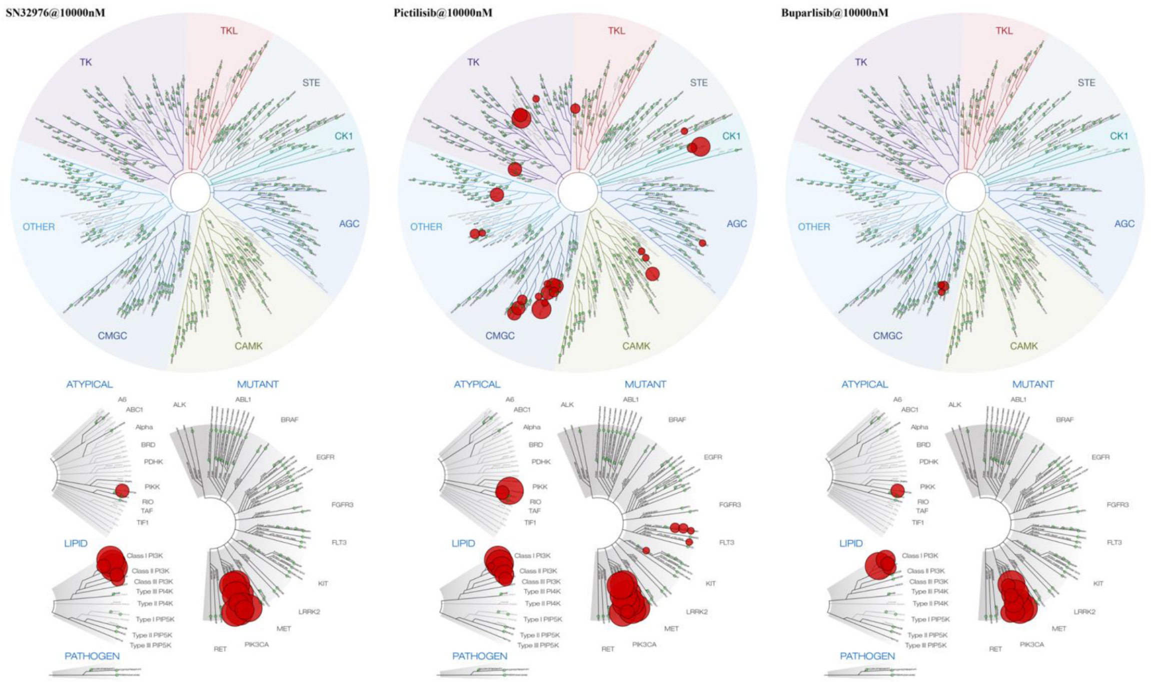

Supplementary Figure 2: Kinase dendrogram plots to visualize kinase interactions in the presence of 10 µM concentrations of each PI3K inhibitor. Kinases that bind at >80% are marked with red circles, with larger circles indicating higher-affinity binding. Images generated using TREE*spot*™ Software Tool and reprinted with permission from KINOME*scan*®, a division of DiscoveRx Corporation, ©DISCOVERX CORPORATION 2010.

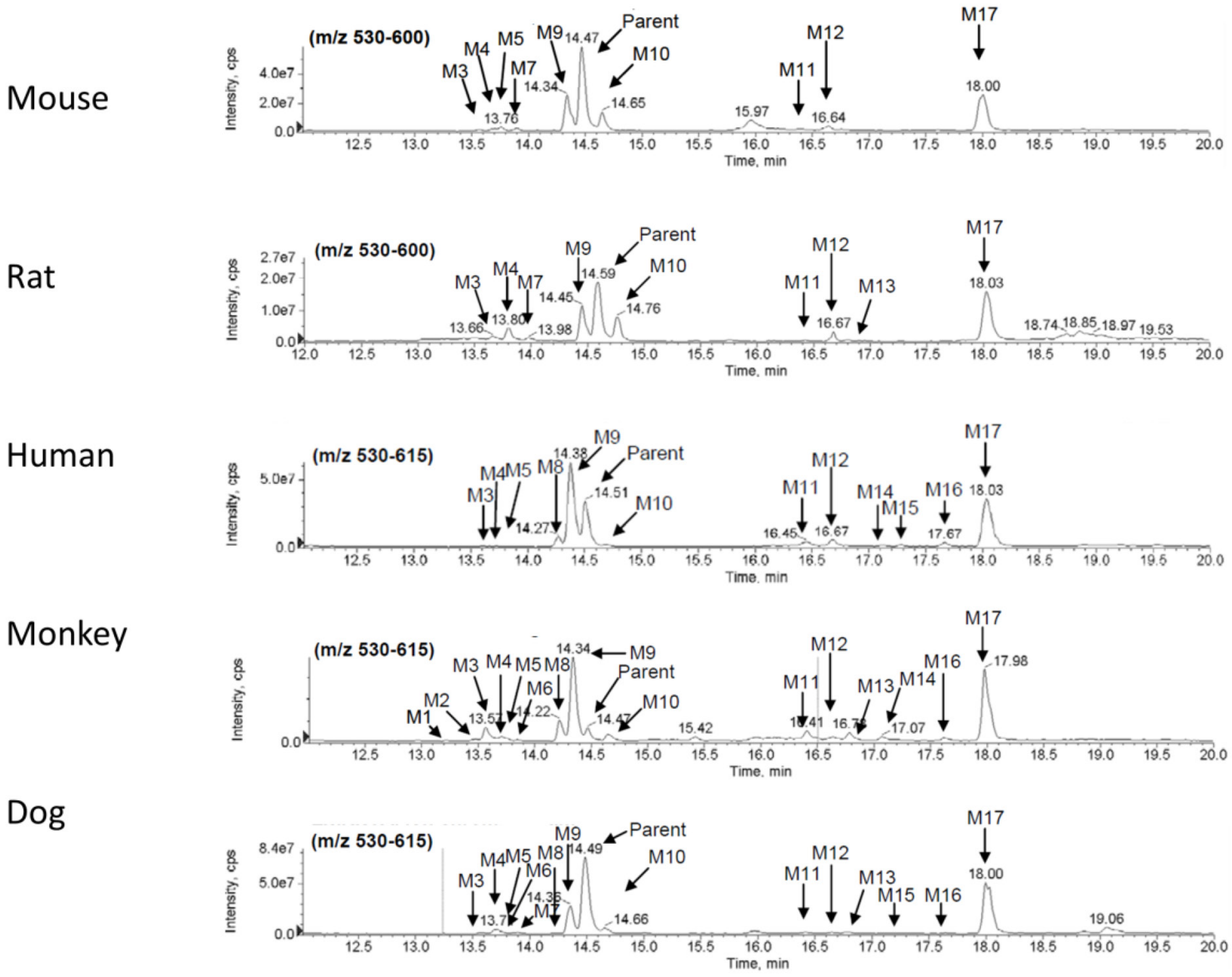

Supplementary Figure 3: Extracted ion chromatographs of parent SN32976 and metabolites with m/z of 530-600 or 530-615 in liver microsomes from multiple species. Microsomes were incubated with 10 µM SN32976 and 2 mM NADPH for 1 h. Peak intensity is displayed in counts per second (cps).

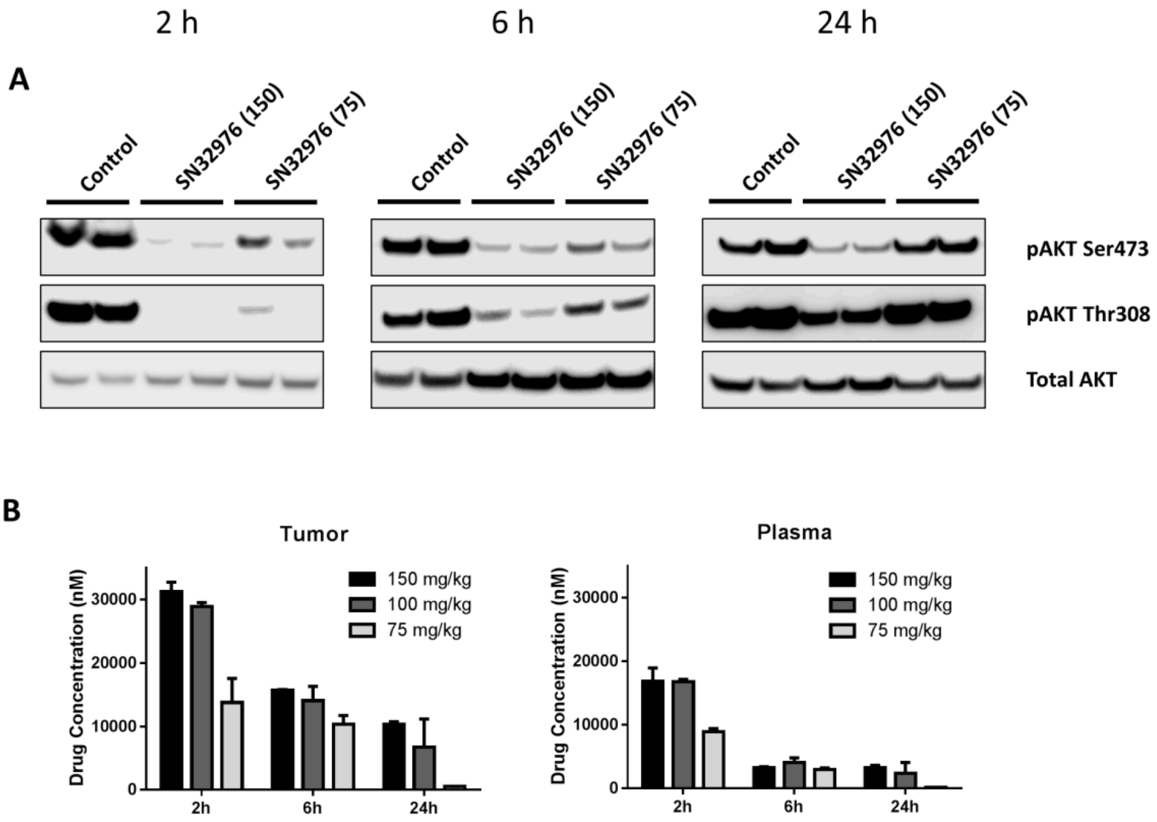

Supplementary Figure 4: Pharmacokinetic-pharmacodynamic response of SN32976 in mice with U-87 MG tumors. Mice with U-87 MG tumors were treated with a single dose of SN32976 at multiple dose levels. **(A)** pAKT and total AKT expression in U-87 MG tumors at multiple timepoints after dosing. **(B)** U-87 MG tumor and plasma drug concentrations of SN32976 at multiple timepoints after dosing. Bars represent the mean and standard error of n=2. The bars above the blots in **(A)** indicate that duplicate cultures were tested at each dose level.

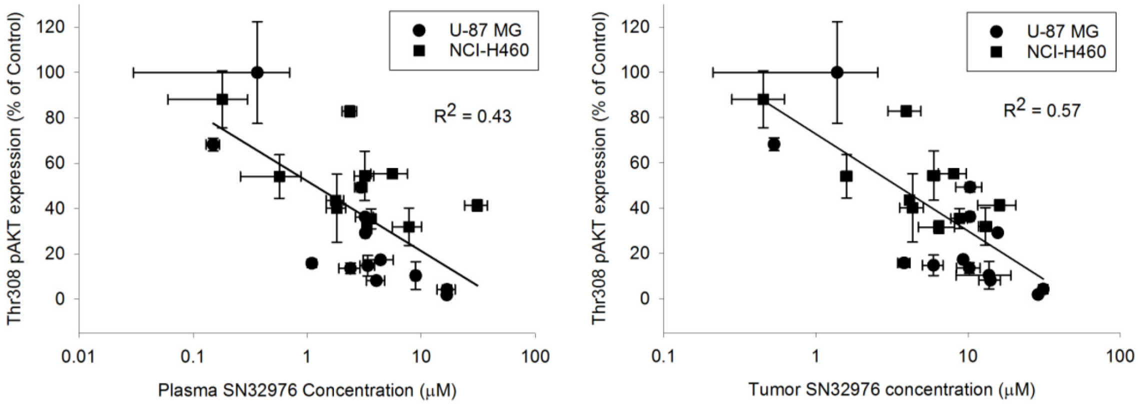

Supplementary Figure 5: Correlation between tumor Thr308 pAKT expression and plasma or tumor SN32976 concentrations. Mice with U-87 MG or NCI-H460 tumors were treated with a single dose of SN32976 at multiple dose levels. Plasma and tumor were collected at 2, 6 or 24 h after treatment. Symbols represent the mean and standard error of n=2.
